# The sound of engagement: assessing the feasibility and acceptability of an AI-generated personalized podcast as a between-session resource for therapy

**DOI:** 10.3389/fdgth.2026.1821642

**Published:** 2026-06-10

**Authors:** Emily E. Peake, Mackenzie S. Swirbul, Michael J. Rodio, Aarthi Padmanabhan

**Affiliations:** Talkspace, New York, NY, United States

**Keywords:** AI in mental health, between-session activities, blended care, feasibility and acceptability, psychotherapy engagement

## Abstract

**Introduction:**

Consistent client engagement with between-therapy session activities (i.e., homework) is a strong predictor of positive psychotherapy outcomes. Because traditional homework activities aren't always tailored to the individual, they can fail to provide the support patients need to translate session insights into real-life change. The integration of digital tools into weekly psychotherapy presents an opportunity to overcome these limitations through personalized blended care.

**Methods:**

This study describes the development and initial validation of Talkcast, a novel AI-powered tool that enables therapists to generate personalized audio discussions (“episodes”) to reinforce concepts from prior therapy sessions. We examined its feasibility (provider/client usage) and acceptability (provider/client quality ratings) across a five-month period (April–September 2025) on a large-scale virtual therapy platform. We also used Linear Mixed-Effects Models to evaluate associations between Talkcast usage and client engagement metrics (live sessions, provider messages, client messages).

**Results:**

Of 6,032 active providers, 46.27% generated a Talkcast, and of 105,317 active clients, 12.97% received one, with 52.70% of recipients opening it. Over 90% of sent Talkcasts rated by providers were “Helpful,” primarily for “Session Reinforcement/Recap”. Similarly, 76.02% of client ratings were “Helpful,” citing “Personalization & Relevance” and “Specific Activities/Tools”. The analysis found a significant positive association between opening a Talkcast and higher overall platform engagement in all measured metrics (all *p* < 0.001). Qualitative feedback highlighted the need for improved content specificity and AI authenticity.

**Discussion:**

Talkcast demonstrated promising feasibility and high acceptability among both providers and clients. Its positive association with key client engagement metrics suggests that Talkcast is a well-integrated blended care tool used by engaged clients to reinforce the therapeutic process. Future work should focus on establishing causality and refining the AI's content generation.

## 1 Introduction

Between-session engagement is an important component of successful psychotherapy (1). Structured activities assigned by the therapist, traditionally termed “homework,” account for significant variance in treatment outcomes (2, 3). These assignments enhance therapeutic outcomes by providing clients with tangible opportunities to apply therapeutic insights and skills to real-world challenges, thus catalyzing actionable change in their daily lives. However, the benefit of these activities is often contingent upon a client's ability to sustain therapeutic momentum in the interval between therapy appointments. Therapy clients may need continuous reinforcement and support to ensure that therapeutic concepts remain accessible and actionable; in turn, this reinforcement and support help prime the therapy client for more intensive skill application and more productive subsequent sessions.

Despite the established value of between-session work in structured therapeutic modalities, such as Cognitive Behavioral Therapy (CBT) and exposure-based treatments (1–3), traditional methods for facilitating between-session engagement such as paper-based assignments (e.g., worksheets), digital lessons that accompany therapy, and self-guided mobile applications or diary logging (4) often encounter barriers to adherence. These assignments can feel disconnected from the live session and lack personalized support, leading to client frustration and non-completion (5, 6).

Integrating digital between-session activities with regular psychotherapy, known as blended care (7), has shown promise in overcoming traditional barriers to between-session engagement (8) by providing real-time prompts and interactive feedback that sustain the therapeutic alliance outside of the clinic (9). When digital activities are paired with talk therapy, clients exhibit higher rates of between-session activity completion and experience significant symptomatic reduction (2, 10, 11). For example: Tailored, AI-enabled therapy support tools, such as chatbots, have been associated with improved session attendance and reduced attrition by providing real-time conversational support and timely intervention reminders (11). However, implementation of these tools is limited. While digital interventions are most effective when actively supported by a licensed provider (12, 13), clinicians rarely have the bandwidth to engage with clients between scheduled sessions or to provide personalized reinforcement of session content, creating a clinical resource gap.

To address this, therapists can leverage digital technology to develop and deliver personalized, clinician-vetted content to support clients outside of the therapy session, and to ensure that between-session reinforcement remains rooted in the specific therapeutic alliance without requiring additional time from the provider. This study introduces and evaluates Talkcast, a novel AI-powered tool designed for between-session reinforcement of therapeutic concepts. The tool aims to provide a clinically vetted touchpoint for clients, especially those who are motivated to engage with therapy outside of the live session. The ultimate goal is to reinforce therapeutic concepts and key discussion points from prior therapy sessions.

The purpose of this study is to describe the development, deployment, and initial validation of Talkcast on a large-scale online therapy platform. We examine its feasibility (provider and client usage), acceptability (client and provider quality ratings), and explore the relationship between Talkcast utilization and key client engagement metrics.

## 2 Materials and methods

Talkcast was developed and implemented within Talkspace. Talkspace is a HIPAA-compliant online therapy platform that matches individuals (referred to here as members) with licensed mental health care providers (referred to here as providers) in their U.S. state of residency. Members, who may be able to access Talkspace via their insurance provider, employer, or independently, may choose to connect with their provider via asynchronous text, live text, live video, or live audio messaging. Talkspace's offerings include specialized individual therapy, couples therapy, and psychiatry.

At the time of the current analysis, Talkcast had not yet been implemented for couples therapy, psychiatry, and certain live-session plans. Therefore, the analysis includes only Talkcasts generated from eligible individual therapy sessions.

### 2.1 Ethical considerations

All data were collected as part of ongoing Talkspace business operations, and the study was deemed exempt from further review by an Institutional Review Board. This was a retrospective data analysis and as such neither therapists nor clients were compensated for their participation or incentivized to provide feedback.

### 2.2 Talkcast overview

The Talkcast feature was developed using Azure OpenAI and Eleven Labs with a HIPAA-compliant infrastructure to securely process session data from live video transcripts, live audio transcripts, live messaging, and asynchronous messaging and generate a podcast style audio file. Talkspace's AI Governance Committee, a group comprised of clinical, legal and regulatory compliance, product, engineering, and industry experts, reviewed the Talkcast feature during the design phase to ensure it met Talkspace's standards for usability, transparency, explainability, and privacy. Various prompts were iteratively tested and refined to optimize for clinical appropriateness standards. During a beta testing period, clinicians tested Talkcast and provided feedback that was used to iterate on the model prior to a wider release. The wider release was presented to therapists live to facilitate understanding of the use, safety, and controls around the process. Throughout the rollout, we emphasized that the feature is an optional tool for them to use. There were no performance metrics or incentives for clinicians to use the feature.

To generate a Talkcast, the therapy transcript was first redacted using a combination of commercially available (Amazon Web Services Glue's entity detection) and open-source (python library “scrubadub”) redaction software to ensure that all protected health information (PHI) and personal identifying information (PII) are removed. As part of ongoing research and machine learning work, transcript quality and redaction accuracy was monitored and improvements were made to the redaction process.

Prior to using Talkcast in a given therapy room, both the licensed provider and individual member consented to the use of the therapy session content for Talkcast episode generation. Following a session, providers could generate a Talkcast if and when they felt that a Talkcast episode would be clinically appropriate for the client. The provider could also choose to write an additional “topic” to be included, e.g., a concept that the provider did not have time to discuss with the therapy client or something that they did discuss but want to emphasize more. The Talkspace platform then sent the de-identified transcript data and the prompt to the Azure API. The prompt included instructions for the GPT model to generate a podcast script based on the themes and topics covered in the therapy transcript provided. When Talkcast was initially introduced, the API then sent the script to the Eleven Labs API, which converted the text-based script to AI-generated “voices”. Then the API returned the script and the Talkcast episode to the Talkspace platform for the provider to review and, optionally, to share with their client.

Based on early feedback, the Talkcast feature was updated to allow providers to edit Talkcast scripts before generating the audio. Beginning July 10, 2025, the Talkcast feature first returned a draft episode script to the therapist to review and edit. After the provider edited and/or approved the script, the provider could opt to generate the audio of the Talkcast via the Eleven Labs API. The APIs did not retain any data.

Following each Talkcast episode generation, providers were prompted to provide optional feedback on the Talkcast episode with the options of “Not good,” “Just okay,” or “Helpful”. If a provider selected “Not good,” the provider could then identify reasons for the negative rating from three options: “I wasn't satisfied with the content,” “It doesn't fit into my workflow,” and “Episode length”. If the provider selected “Just okay,” the provider could then identify reasons for the rating from three options: “I wasn't satisfied with the content,” “It doesn't fit into my workflow,” and “Episode length”. If the provider selected “Helpful,” indicating a positive rating, the provider could then identify reasons for the rating from three options: “Quality content,” “It fits well into my workflow,” and “Episode length”. For each rating, providers could select multiple reasons and could also provide additional details to their feedback selection within a free response section. Feedback was stored and evaluated by the Talkspace product team for further refinements of the feature, when required.

After listening to a Talkcast, clients were also prompted to provide optional feedback on the Talkcast with the options of “Not good,” “Just okay,” “Helpful,” or “I didn't listen to it”. If “I didn't listen to it” was selected, no additional feedback prompts were presented. If “Not good” was selected, clients could then identify reasons for the negative rating from three options: “Topic wasn't relevant,” “It's not something I enjoy doing,” and “Episode length”. If “Just okay” is selected, clients could then identify reasons for the rating from three options: “Topic wasn't relevant,” “It's not something I enjoy doing,” and “Episode length”. If “Helpful” was selected, indicating a positive rating, clients could then identify reasons for the rating from three options: “Felt relevant to me,” “Insightful content,” and “Helpful activity between sessions”. For each rating, clients could select multiple reasons and could also provide additional details to their feedback selection in a free-response section. Clients were also given the option to opt out of receiving another Talkcast episode at this stage.

Talkspace trained providers on, and granted providers access to, Talkcast in a stepwise feature rollout. Talkcast was launched to the entire provider network for providers to implement for individual client sessions in April 2025.

Data was captured through integrated platform mechanisms as part of standard clinical quality monitoring. While responses are identifiable within the system to facilitate healthcare operations, they were de-identified for the purpose of this analysis.

### 2.3 Clinical risk mitigation

Talkcast as a feature included multiple levels of risk mitigation. First, the large language model (LLM) prompt included model constraints to minimize hallucination and content that would not be clinically useful. Second, the feature was designed with the clinician in the loop, providing clinical gatekeeping to determine if the content was safe for the client to consume. Third, there was an option for the client to pre-emptively opt out of using Talkcast or to later opt out at any point. Finally, there was aggregate feedback monitoring to identify systematic inaccuracies and clinician preferences.

The platform operated on a decentralized clinical responsibility model. Because Talkcast was a tool provided to licensed clinicians, escalation occurred at the point of review: if a draft contained inaccuracies (e.g., misaligned focus or hallucinations), the clinician was ethically and procedurally obligated to edit or discard the draft. Monitoring of feedback was used to tune the underlying models to reduce provider burden and review fatigue.

### 2.4 Data and analyses

**Figure 1:**
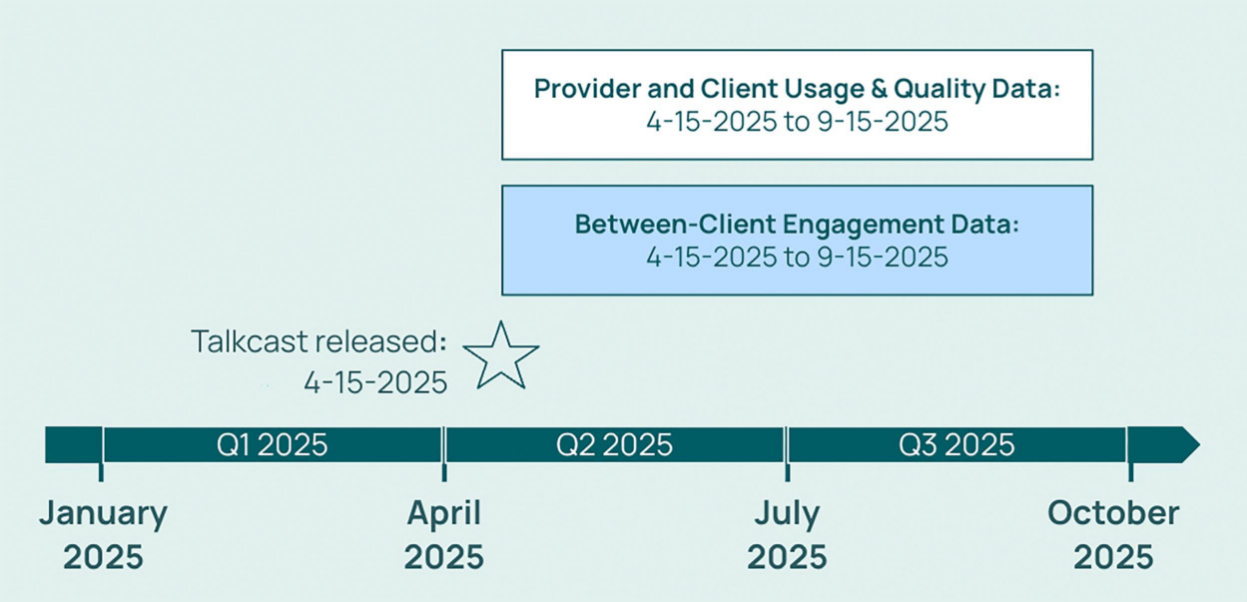
Data collection and analysis periods.

We first examined provider- and client-level Talkcast usage, overall client engagement with the Talkspace platform, and client- and provider-rated Talkcast quality using descriptive statistics and *t*-tests. Key dates and timelines used for data collection and analysis are shown in Figure 1.

#### 2.4.1 Talkcast usage

We analyzed providers’ Talkcast usage (ie, the number and percentage of providers who generated, sent, and or provided feedback about Talkcasts as compared to total providers) and clients’ Talkcast usage (ie, the percentage of clients who received, opened, and or provided feedback about Talkcasts as compared to total clients).

#### 2.4.2 Client engagement

We then analysed clients’ engagement with therapy. We included clients who had an active account with one or more calls or messages during the study period and whose provider had sent at least one Talkcast. We utilized Generalized Linear Mixed Models (GLMM) with a Negative Binomial distribution to evaluate associations between Talkcast use and three distinct engagement metrics: counts of live sessions, provider messages, and client messages. This approach was selected to account for the overdispersed count nature of the engagement metrics. The analysis compared three groups: Not Sent (Baseline), Not Opened, and Opened. Models were controlled for provider-level random effects, client tenure (the amount of days they had been on the Talkspace platform), and baseline activity (calls or messages 30 days prior to the study period).

#### 2.4.3 Provider-rated Talkcast quality

We examined the quantitative and qualitative aspects of providers’ quality feedback. We used Google Gemini 3 Flash (running on the Paid tier) to analyze the qualitative aspects of the data. We provided the following standardized prompt template in batches organized by user rating (e.g., all “Helpful” responses were processed together):

*Analyze the following free-text feedback responses from therapists who rated the Talkcast tool as [RATING]. Identify three to five overarching themes, within those, identify and define specific subthemes. For each subtheme, provide a count and percentage of how many responses apply. Present the response in a table*.

To ensure accuracy of the themes, the authors manually reviewed all anonymized, de-identified free-responses to verify that the themes were contextually appropriate. Where the LLM generated overlapping or redundant themes, the authors merged these into a single category to ensure mutual exclusivity. Where appropriate, the authors re-ordered the themes for consistency.

Data is presented at the feedback level (e.g., providers may have supplied feedback for more than one Talkcast) and each response was allowed to be assigned to multiple themes.

#### 2.4.4 Client-rated Talkcast quality

Similarly, we examined the quantitative and qualitative aspects of clients’ quality feedback. Again, we used Google Gemini 3 Flash (running on the Paid tier) to analyze the qualitative aspects of the data. Similarly, we provided the following standardized prompt template in batches organized by user rating (e.g., all “Helpful” responses were processed together):

*Analyze the following free-text feedback responses from clients who rated the Talkcast tool as [RATING]. Identify three to five overarching themes, within those, identify and define specific subthemes. For each subtheme, provide a count and percentage of how many responses apply. Present the response in a table.*

To ensure accuracy of the themes, the authors manually reviewed all free-responses to verify that the themes were contextually appropriate. Where the AI generated overlapping or redundant themes, the authors merged these into a single category to ensure mutual exclusivity. Where appropriate, the authors re-ordered the themes for consistency. Data is presented at the feedback level (e.g., clients may have supplied feedback for more than one Talkcast) and each response was allowed to be assigned to multiple themes.

## 3 Results

Between April 15th, 2025 and Sept 15th, 2025 (Figure 1), 2,791 unique providers generated 39,982 Talkcasts and sent 28,165 Talkcasts to 13,661 unique clients.

### 3.1 Usage

#### 3.1.1 Providers

Out of 6,032 active providers on the platform between April and September, 2,791 (46.27%) providers generated at least one Talkcast. Of those, 1,301 (46.61%) providers sent at least one Talkcast. 1,103 providers who sent a Talkcast provided feedback. 839 providers provided feedback about Talkcasts that they did not send. Of the providers who sent at least one Talkcast, the average number of Talkcasts sent was 21.65 (SD = 50.14; Median = 4). These providers had an average caseload of 13.79 clients (SD = 18.54; Median = 6). Providers sent Talkcasts to an average of 10.56 clients (SD = 18.71; Median = 3) in their caseload.

#### 3.1.2 Clients

Of the 105,317 active clients on the platform during the evaluation time period, 13,661 (12.97%) clients received at least one Talkcast and 7,199 (52.70%) clients who received a Talkcast opened at least one Talkcast. 3,028 (42.06%) clients provided feedback for at least one Talkcast. Of the clients who opened at least one Talkcast, the average number of Talkcasts received was 2.55 (SD = 2.31; Median = 2) and opened was 2.35 (SD = 2.21; Median = 2).

### 3.2 Engagement with therapy

#### 3.2.1 Between-participant analysis: Talkcast usage and engagement with therapy

As shown in Table 1, usage of Talkcasts was associated with significantly higher counts of calls and messages. Compared to the Not Sent group, therapy rooms where a Talkcast was Opened saw the largest lift, with a 24% increase in calls (IRR = 1.24, *p* *<* *.001*), a 40% increase in provider messages (IRR = 1.40, *p* *<* *.001*), and a 47% increase in client messages (IRR = 1.47, *p* *<* *.001*). Notably, the Not Opened group did not show a statistically significant increase in calls (IRR = 0.98, *p* *=* *.49*) or provider messages (IRR = 1.04, *p* *=* *.11*) relative to the baseline, and was associated with a significant 8% decrease in client messages (IRR = 0.92, *p* = .003). This suggests that engagement with therapy was associated with the client's active usage of the Talkcast rather than its delivery alone.

**Table 1:** Fixed effects of Talkcast utilization and engagement with therapy.

Outcome Metric	Predictor	IRR	95% CI	Est. (SE)	z	*p*
Live Calls	(Intercept)	–	–	1.55 (0.01)	114.27	<.001
	Not Opened	0.98	[0.95, 1.01]	−0.02 (0.01)	−1.13	.493
	Opened	1.24	[1.21, 1.28]	0.22 (0.01)	14.84	<.001
	Tenure (Z)	1.03	[1.02, 1.04]	0.03 (0.01)	5.98	<.001
	Baseline Calls (Z)	1.32	[1.31, 1.33]	0.28 (0.01)	53.27	<.001
Provider Msg	(Intercept)	–	–	2.53 (0.02)	129.26	<.001
	Not Opened	1.04	[1.00, 1.08]	0.04 (0.02)	2.02	.108
	Opened	1.40	[1.35, 1.45]	0.34 (0.02)	18.44	<.001
	Tenure (Z)	1.04	[1.02, 1.05]	0.04 (0.01)	5.33	<.001
	Baseline Msg (Z)	1.29	[1.27, 1.31]	0.26 (0.01)	32.34	<.001
Client Msg	(Intercept)	–	–	2.13 (0.03)	83.44	<.001
	Not Opened	0.92	[0.87, 0.96]	−0.09 (0.03)	−3.27	.003
	Opened	1.47	[1.39, 1.55]	0.39 (0.03)	14.29	<.001
	Tenure (Z)	1.09	[1.07, 1.11]	0.09 (0.01)	8.23	<.001
	Baseline Msg (Z)	1.75	[1.69, 1.81]	0.56 (0.02)	29.50	<.001

*N* = 16,708 observations nested within 2,249 providers. (ref) = Not Sent group. All continuous covariates (Baseline, Tenure) were standardized (Z-scores) prior to modeling. IRR, incident rate ratio (e^Estimate^).

The robustness of these fixed effects is supported by the model diagnostics and random effect structures summarized in Table 2. Across all outcomes, the inclusion of provider-level random intercepts accounted for significant variance, justifying the mixed-effects approach to control for provider-specific communication styles. Furthermore, the dispersion parameters (*Θ*) indicate that the Negative Binomial framework successfully addressed the overdispersion inherent in the engagement data.

**Table 2:** Random effects and model diagnostics.

Metric	Random Effect Var (*σ*^2^)^a^	Dispersion (*θ*)	AIC	logLik
Live Calls	0.069	4.326	84,927	−42,457
Provider Messages	0.287	1.871	125,021	−62,503
Client Messages	0.287	0.802	112,003	−55,995

a Variance attributed to the random intercept of Provider ID.

However, the Opened group significantly outperformed the Not Opened group across all metrics (*p* < .001), demonstrating that utilization of at least one Talkcast co-occurred with significantly higher levels of session attendance and messaging activity.

#### 3.2.2 Covariates and random effects

Baseline engagement was a strong and significant predictor in all models (*p* *<* *.001*), confirming that the models successfully accounted for pre-existing activity levels. Platform tenure also showed a positive association with messaging volume *(p* *<* *.001*). The random intercept of provider remained significant, justifying the mixed-effects approach to control for inherent differences in provider communication styles.

#### 3.2.3 Timing and delivery latency

We conducted a sub-analysis of the Talkcast sent group (Table 3) to examine the timing of the first Talkcast delivery (the latency between session end and Talkcast creation). For live calls, we found a significant negative association between post-live-session latency and the number of subsequent live sessions. Conversely, for provider messages, a slight positive association was found, suggesting that providers who took more time to deliberate before sending a Talkcast engaged in higher total messaging volumes.

**Table 3:** Sub-analysis of the impact of Talkcast timing on engagement.

Metric	Predictor	Estimate (Log)	SE	z	*p*
Calls	Timing_Z	−0.028	0.007	−4.16	<.001
Prov. Msg	Timing_Z	0.026	0.008	3.27	.001
Client Msg	Timing_Z	0.042	0.012	3.51	<.001

#### 3.2.4 Robustness checks

To ensure the high-count “power users” were not biasing the results, a sensitivity analysis was conducted by excluding the top 1% of observations based on Pearson residuals. The fixed effect for the Opened group in the Client Messages model remained stable (Original Estimate: 0.39; Filtered Estimate: 0.38), confirming that the findings are robust across the broader group.

### 3.3 Quality

#### 3.3.1 Therapist ratings

**Figure 2:**
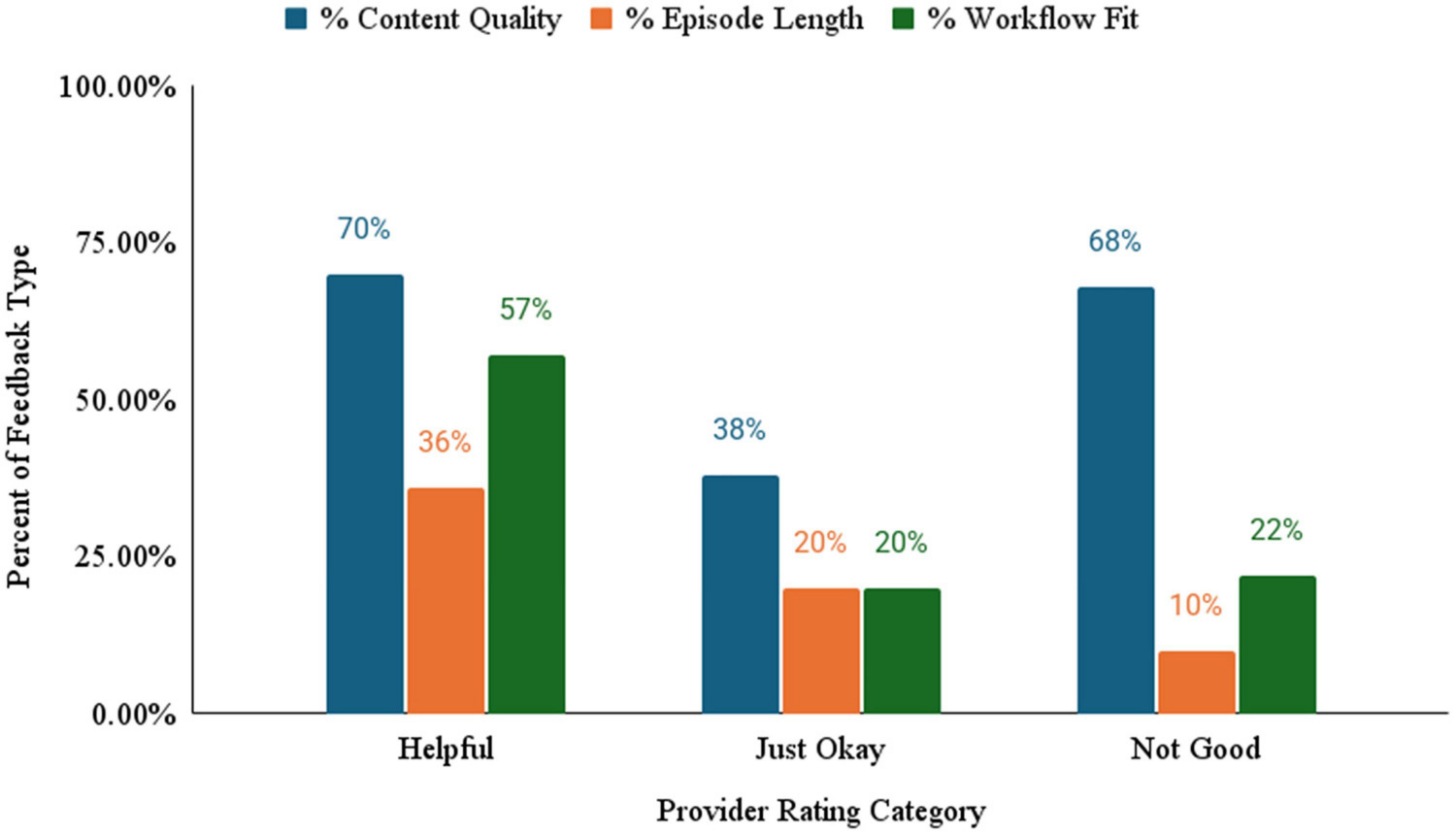
Provider feedback across categories.

**Figure 3:**
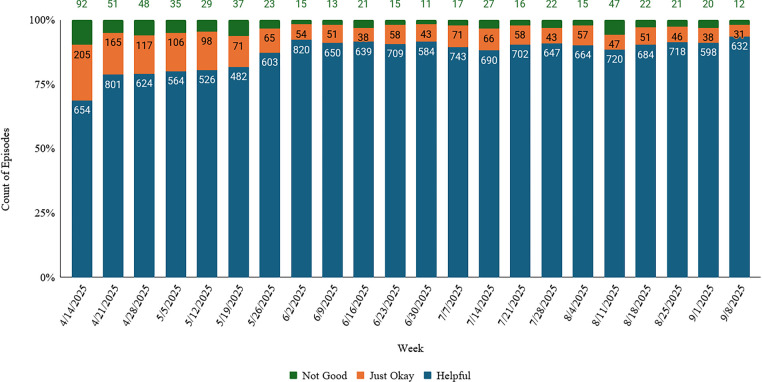
Provider ratings by week.

29.56% of Talkcasts generated were not sent, indicating that the clinician-in-the-loop safety feature was actively filtering out clinical inaccuracies. Therapists primarily rated the Talkcasts that were selected to be sent to clients as “Helpful,” (92.47%), whereas for Talkcasts that were generated but not sent, therapist ratings were lower and more evenly distributed among “Helpful” (33.56%), “Just Okay” (34.93%), and “Not Good” (31.51%) (Table 4). This marked difference suggests that the high helpfulness rate for delivered Talkcasts is a reflection of provider pre-selection, where therapists were able to act as clinical gatekeepers to screen out less helpful content prior to sending, in line with expected “clinician-in-the-loop” behavior. Across the rating categories, the majority of providers’ feedback was related to the content quality, followed by workflow fit and episode length (Figure 2). Beginning when the product was fully introduced in April 2025, ratings were primarily “Helpful” (69%); this proportion trended higher over time through September (max = 94%; Figure 3).

**Table 4:** Therapist ratings of sent vs. not sent Talkcasts.

Feedback	Sent		Not sent	
	*n*	%	*n*	%
Helpful	13,917	92.47%	540	33.56%
Just Okay	1,020	6.78%	562	34.93%
Not Good	113	0.75%	507	31.51%
Total	15,050	100.00%	1,609	100.00%

Therapist free-text feedback was analyzed and grouped into themes (Table 5). The analysis revealed that when the Talkcast feature was rated as Helpful (*n* = 419), its primary strength was Clinical Utility and Reinforcement, which accounted for nearly 90% of the positive feedback. Specifically, therapists highly valued the feature for providing Session Reinforcement/Recap (39.40%), offering Actionable Tools/Homework for clients (21.00%), and demonstrating strong Relevance & Clinical Fit (16.20%). Furthermore, feedback that praised the Positive/Supportive Tone and Format/Concept Approval (both 6.20%) confirmed positive engagement with the format.

**Table 5:** Therapist feedback thematic analysis.

Talkcasts Sent to Clients			
Rating: Helpful (*n* = 419)			
Theme Category	Specific Theme	Count	Percentage
Clinical Utility & Reinforcement	Session Reinforcement/Recap (Reinforces, summarizes, and adds support to session content)	165	39.40%
	Actionable Tools/Homework (Provides exercises, coping skills, and concrete next steps for clients)	88	21.00%
	Relevance & Clinical Fit (Content is appropriate, on-topic, well-adapted to client issues/goals)	68	16.20%
	Introduction of New Tools/Insight (Adds new skills, insights, or expounds on topics beyond the session)	39	9.30%
Format & Engagement	Personalization (Using therapist's/client's name, personalized to conversation)	26	6.20%
	Positive/Supportive Tone (Uplifting, encouraging, compassionate, validating)	26	6.20%
	Format/Concept Approval (Intriguing, cool, great idea, awesome tool, “client likes this”)	25	6.00%
Areas for Improvement/Caveats	Length/Repetitiveness (Too long, repetitive, need for shorter episodes)	39	9.30%
	Technical Issues (Stops midway, needs transcript, mispronunciation of name)	18	4.30%
	AI/Authenticity/Naming Issues (Concerns about AI tone, naming therapist/client in script)	9	2.10%
	Workflow/Time Issues (Hard to wait for generation, hard to find time to review)	7	1.70%
Rating: Just Okay (*n* = 300)			
Content Depth & Specificity	Too General/Basic/Vague (Not personalized, elementary, repetitive with generic concepts)	73	24.30%
	Lack of Depth/Insufficient Detail (Didn't explain techniques, ended abruptly, needed more information)	49	16.30%
	Content Inaccuracies/Omissions (Missed key topics, misquoted/misunderstood session details, wrong focus)	42	14.00%
Format and AI Experience	Length/Repetitiveness (Too long, repetitive scripting, drawn out intro/outro)	76	25.30%
	Desire for Editing/Control (Wish to edit script, change word choices, save previous versions)	26	8.70%
	AI/Authenticity/Naming Issues (AI sounds robotic/cold, AI hosts use therapist/client name or fake stories)	21	7.00%
	Privacy Concerns/Feels Invasive (Feels like strangers are listening, invasion of privacy)	3	1.00%
Workflow and Functionality	Workflow/Time Issues (Takes too long to load/review, need time credit for review)	16	5.30%
	Technical Issues (Cursor spinning, audio cut off, app bugs)	6	2.00%
	Inappropriate Tone/Language (“Challenging” is too harsh, not culturally sensitive)	4	1.30%
	Other/Mixed/Neutral (Satisfied with some, unsure how client will receive)	5	1.70%
Rating: Not Good (*n* = 66)			
Technical/Functionality Issues	Failure to Load/Send/Post (Will not generate, send button broken, unable to scroll to approve, not posting to client room)	26	39.40%
	Audio Cut-off/Incomplete (Cuts off in the middle of content, not fully generating)	8	12.10%
	Inability to Preview/Hear Audio	3	4.50%
Content and Utility	Too Long/Excessive Detail (Over 15 min, too much to review, too much detail)	5	7.60%
	Repetitive/Lack of Variation (Repeats skills, same as last episode)	7	10.60%
	Content Irrelevance/Misaligned Focus (Wrong primary focus, not relevant, missed key topic, misstated fact)	7	10.60%
	Lack of Specificity/Explanation (Did not explain technique, needed better steps/examples)	4	6.10%
	AI Unable to Cope with Client Complexity	1	1.50%
Workflow and Other	Workflow/Compensation (Too much unpaid time to review, not timely to session)	3	4.50%
	Corny/Unprofessional Source (Corny tone, cited IG influencer)	2	3.00%

Talkcasts Not Sent to Clients			
Rating: Helpful (*n* = 91)			
Theme Category	Specific Theme	Count	Percentage
Clinical Utility & Reinforcement	Session Reinforcement/Recap (Accurately reflected, summarized, and reinforced session topics)	26	28.60%
	Added Insight/Clarity (Gave therapist additional ideas, insight, or clear context)	10	11.00%
	Actionable Tools/Homework (Provided a tool/exercise for client practice)	7	7.70%
Format and Client Engagement	Length Issues (Too long, much too long, needed to be shorter)	11	12.10%
	Client Readiness/Consent (Need to discuss with client first, not sure client will take time to listen, fear of overwhelming)	8	8.80%
	Need for Transcript/Alternative Format (Client would like a written transcript, struggles with audio only)	2	2.20%
Content and Accuracy	Content Irrelevance/Misaligned Focus (Focused on secondary topic like perfectionism, not relevant to primary issue)	5	5.50%
	Repetitive/Lacks New Information (Repeated skills, repeated topic from last Talkcast)	5	5.50%
	Accuracy/Clarity Issues (Not accurate to session flow, missed point, technical terms unexplained)	4	4.40%
Technical & Workflow Failures	Failure to Send/Load/Function (Will not send, “wheel kept spinning,” frozen screen, had to manually copy/paste)	10	11.00%
	Workflow Issue (Lack of time to review, need ability to send later)	3	3.30%
Rating: Just Okay (*n* = 124)			
Format and Tone	Too Long/Excessive Fluff (Way too long, wordy, “fluff/rambling,” “too chunky,” will lose attention)	26	21.00%
	Cheesy/Corny/Artificial (Lacks sincerity, sounds mechanical, “icky feeling,” “hokey,” non-authentic dialogue)	19	15.30%
	Dislike of Podcast/Hosts/Format (Prefers text/recap, finds back-and-forth odd, intrusive characters)	15	12.10%
	Tone is Inappropriate (Too lighthearted/dismissive for serious topics, condescending, not validating)	4	3.20%
Content and Relevance	Content Irrelevance/Misaligned Focus (Missed key topic/suggestion, focused on wrong issue, not suitable for client stage)	19	15.30%
	Lacks Depth/Specificity (Too vague/basic, needs more science, didn't explain exercise, not creative enough)	16	12.90%
	Repetitive/Lack of Variation (Repeats same message, same skills like Window of Tolerance, already practiced)	12	9.70%
	AI Authenticity is Offensive/Misleading (AI trying too hard to relate culturally, uses copyrighted terms, fake stories)	8	6.50%
Technical & Workflow Failures	Failure to Send/Load/Edit (Wouldn't send, couldn't edit input/script, cut off mid-content, couldn't load)	12	9.70%
	Workflow/Time/Compensation (Takes too long to review, need to ask client consent first, uncompensated time burden)	7	5.60%
Rating: Not Good (*n* = 128)			
Technical/Functionality Issues	Failure to Load/Send/Complete Audio (Will not generate, send button broken, spinning wheel, audio incomplete/crashed)	36	28.10%
	Inability to Edit/Access Past Version (Couldn't edit, couldn't go back to original version)	4	3.10%
Format and Tone Rejection	Corny/Cheesy/Artificial Tone (Too corny, childish, too upbeat, sounds fake, “ick”)	14	10.90%
	Conceptual Rejection of Podcast Format (Dislike of “podcast idea,” “weird talk show,” “silly,” “mockery”)	13	10.20%
	AI Pretending to be Human (Trivializes experience, demeaning, confusing “hosts,” “life coach” title)	12	9.40%
	Creepy/Invasive/Confidentiality Fear (Feels like someone is listening, intrusive, non-confidential)	6	4.70%
Content and Relevance	Content Irrelevance/Inaccuracy (Totally ignored topic, based on old info, hallucinations, combined separate issues)	16	12.50%
	Lack of Depth/Specificity (Vague, superficial, no actual exercise, no real examples, too basic)	12	9.40%
	Length/Repetitiveness (Way too long, too short for content, redundant, repetitive skills)	10	7.80%
Other/Unique Concerns	Ethical/Billing Concern (19-minute audio is a billable intervention, should not say therapist prepared)	2	1.60%
	Wrong Client Status/Name (Sent to client not yet seen, used wrong client name, used therapist name as client)	2	1.60%
	Inappropriate Examples/Sources (Wrong therapeutic technique, celebrity example inappropriate, inappropriate for high intelligence)	3	2.30%

#### 3.3.2 Client ratings

**Figure 4:**
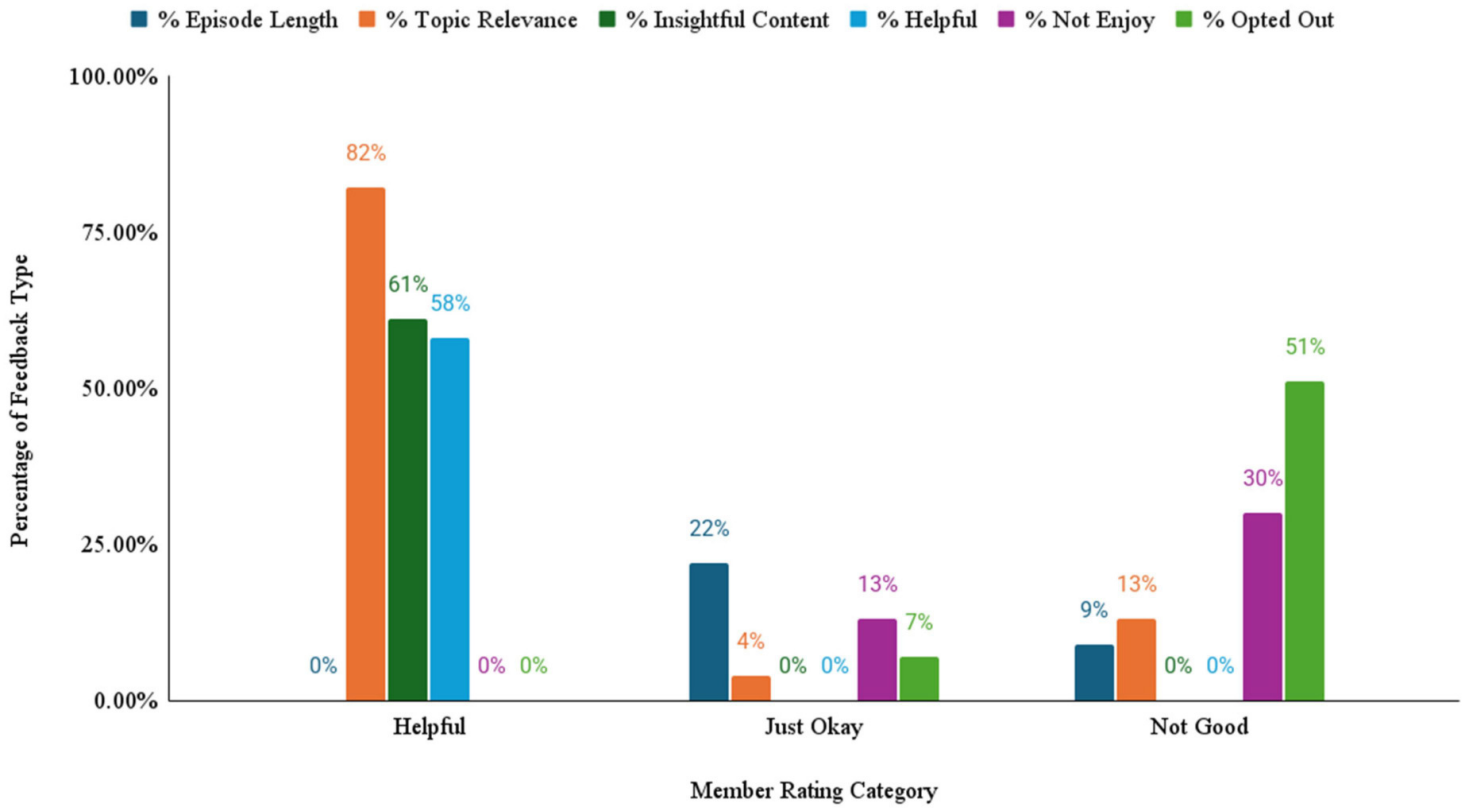
Client feedback across categories.

**Figure 5:**
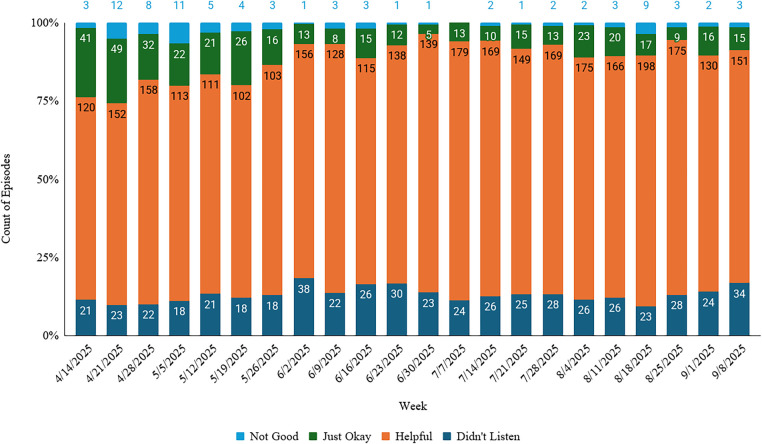
Client ratings by week.

Clients primarily rated the Talkcasts that were opened as “Helpful” (76.2%) followed by “Didn't Listen,” (12.2%) “Just Okay,” (9.87%) and “Not Good” (1.91%) (Table 6). These proportions reflect a total of 4,074 rating events provided by 3,028 unique clients, which represent the subset of clients who chose to submit feedback (42.06% of those who opened a Talkcast). The feedback related to “helpful” ratings was “helpful,” “topic relevance,” and “insightful content”. The feedback related to “Just okay” was “episode length,” “topic relevance,” “not enjoy,” and “opted out”. The feedback related to “Not Good” was “episode length,” “topic relevance,” “not enjoy,” and “opted out” (Figure 4). Beginning when the product was introduced in April 2025, the proportion of ratings was primarily “helpful” (66%) and the proportion trended higher over time through September (max = 84%; Figure 5).

**Table 6:** Client ratings of Talkcasts.

Feedback	Count	%
Didn't Listen	497	12.20%
Helpful	3,097	76.02%
Just Okay	402	9.87%
Not Good	78	1.91%
Total	4,074	100.00%

Client free-text feedback was analyzed and grouped into themes (Table 7).The client feedback affirmed the “Helpful” category (*n* = 384). Clients highly valued the Personalization & Relevance of the content, specifically reporting that it was tailored to their needs (26.00%) and provided crucial Session Reinforcement/Recap (18.80%). The clients commented that it offered Specific Activities/Tools (21.40%) and was vital In-Between Session Support (16.40%). Conversely, the most critical negative feedback focused on issues of authenticity, with clients finding the fake stories and anecdotes to be Inauthentic/Creepy (33.30% in the “Not Good” category), and demanding that the content be less generic and more detailed (30.60% in the “Just Okay” category).

**Table 7:** Client feedback thematic analysis.

Rating: Helpful (*n* = 384)			
Theme Category	Specific Theme	Count	Percentage
Positive Feedback			
Personalization & Relevance	Content is Highly Tailored/Relevant to session/issues/goals	100	26.00%
	Session Reinforcement/Recap (Summarizes, reiterates, and reminds of session content)	72	18.80%
	Validation and Understanding (Felt heard, seen, validated, understood, normalized, not alone)	28	7.30%
Actionable Utility	Specific Activities/Tools (Gave “homework,” exercises, tangible/achievable steps, grounding)	82	21.40%
	In-Between Session Support (Great tool for support, reminders, and work between sessions)	63	16.40%
	Practical Examples (Used real-life stories/scenarios, clear instructions, easy to apply)	31	8.10%
Format & Engagement	Positive Tone/Encouragement (Uplifting, encouraging, reassuring, inspiring, focused on progress)	21	5.50%
	Digestible Format (Liked podcast style, short, concise, easy to listen to, relatable medium)	41	10.70%
	Ability to Re-Listen (Can go back and listen multiple times/refer back to it)	16	4.20%
Areas for Improvement			
AI Authenticity & Tone	AI Sounded Inauthentic/Creepy (Fake stories, hosts speaking as human, awkward, off-putting, cheesy)	24	6.30%
	Voice/Pacing Issues (Voice needs polishing, spoke too fast, felt like a robot)	11	2.90%
Content & Accessibility	Desire for Transcripts/Notes (Wanted a written version/summary to aid processing/note-taking)	17	4.40%
	Length/Specificity Concerns (Could be longer/more specific about technique, or was too repetitive)	7	1.80%
	Technical/Playback Issues (Stops when screen locks, playback is spotty)	2	0.50%
Rating: Just Okay (*n* = 186)			
Content Depth & Specificity	Lack of Detail/Vagueness (e.g., too short, too high-level, missing explanation of techniques)	57	30.60%
	Repetitive/Excessively Wordy	22	11.80%
	Irrelevant/Inaccurate Content (e.g., missed topics, wrong assumptions, generic)	16	8.60%
Format and AI Experience	AI/Robotic Voices (e.g., disengaging, robotic, monotone)	39	21.00%
	AI Pretending to be Human (e.g., fake stories, “I've been there” anecdotes, cheesy)	36	19.40%
	Uncomfortable with AI/Privacy Concerns	12	6.50%
	Too Much Dialogue/Recapping (e.g., too much intro, excessive cross-confirmation, one host not needed)	12	6.50%
Utility and Functionality	Technical/Playback Issues (e.g., stops when screen locks, inability to fast-forward)	14	7.50%
	Need for Transcript/Pauses (e.g., hard to take notes, too fast, need time for reflection)	13	7.00%
	Lack of Personal Value/Helpfulness (General statements of not being helpful or useful)	10	5.40%
	Prior Method Not Helpful	4	2.20%
Rating: Not Good (*n* = 57)			
Authenticity & Trust	Lack of Consent/Privacy Concerns (Session monitoring, use of private data in AI)	7	12.30%
	AI Pretending to be Human (Uncanny valley, fake stories/anecdotes, disingenuous)	19	33.30%
	AI Voices/Robotic Feel	10	17.50%
Content Value	Lack of Value/Generic Content (Did not contribute anything, vague, surface-level, “word salad”)	19	33.30%
	Repetitive/Already Discussed (Quoting past sessions, techniques already known)	8	14.00%
	Content Inaccuracy/Irrelevance (Missed topics, incorrect details, not specific to the user's issue)	5	8.80%
Format and Length	Too Short/Exposition Too Long (Time spent on intro vs. actual content)	7	12.30%
	Insufficient Detail/Unexplained Exercise	4	7.00%
	Technical Issues (Static, app annoyance)	2	3.50%

## 4 Discussion

This study evaluated the feasibility and acceptability of a novel AI-powered therapeutic tool designed to facilitate between-session reinforcement and examined its associations with client engagement metrics on a large-scale virtual therapy platform. The findings provide initial evidence that personalized, AI-generated audio content serves as a well-received blended care tool that aligns with higher levels of client participation, providing a supplemental touchpoint between therapy sessions without increasing clinician burden.

The deployment of Talkcast demonstrated promising feasibility. Over a five-month period, nearly half of active providers utilized the tool, generating at least one Talkcast, and 13% of active clients engaged with the content. Notably, over half of clients who received a Talkcast opened the file, a rate that compares favorably to published adherence rates for traditional self-reported homework completion (5, 14, 15). This study also demonstrated a high adoption rate among providers and a high level of client curiosity and willingness to engage with a new format. Given the documented barriers to clinician technology adoption (16), such as concerns about time-intensive workflows, the high level of provider engagement suggests that providers perceive Talkcast as a low-burden mechanism for extending clinical reach. The integration of a provider's explicit recommendation with the novelty of a personalized podcast format may have helped drive the willingness to engage and mitigate the typical resistance to adopting new healthcare technologies (10, 17).

By requiring a human review before client delivery, Talkcast ensured that providers could edit or reject any episodes that did not meet clinical standards, preserving quality and provider accountability. From an ethical standpoint, the “human-in-the-loop” model allowed for provider oversight in automated processes. While very few providers reported inappropriate content and high send rates suggested that the generated content generally met provider expectations, the sign-off requirement adhered to recommendations for ethical AI integration (18, 19).

Quality ratings from both providers and clients underscore high levels of acceptability for the final, clinician-vetted Talkcasts, while also generating useful feedback for future product iterations. Over 90% of therapists who gave feedback rated Talkcasts as “helpful”, specifically citing the tool's utility in reinforcing session concepts and clarifying actionable tasks. Furthermore, because therapists screened the content first, the high rating also demonstrates the efficacy of the clinician-in-the-loop model, where providers were able to filter out generated content rated as less helpful. Constructive feedback also highlighted a need for further workflow optimization to reduce administrative friction.

Client feedback further supported the tool's role in providing between-session support, positively noting personalization and relevance. Some constructive feedback from clients focused on issues of AI authenticity and tone, highlighting an important point that technology must maintain a sense of clinical presence without attempting to simulate a replacement for the therapeutic alliance. Furthermore, the variability in content specificity, where some outputs were rated as vague/general, missing key topics, or too generic, indicates that the efficacy of these types of AI tools are dependent on the quality of transcript inputs and precision of prompt engineering.

A positive association was observed between Talkcast usage and broader platform engagement. Clients who opened a Talkcast exhibited significantly higher frequencies of live session and message exchanges with their therapist. While these engagement metrics are established correlates of successful therapy outcomes and strong therapeutic alliance (20), the findings are subject to selection bias. As this was an observational study, it is not possible to disentangle whether the Talkcast drove engagement or if already motivated clients were more likely to receive and/or use the feature. Nevertheless, Talkcast may serve as a resource that supports the existing momentum of active therapy participants or to lower the barrier to active participation by keeping therapeutic goals top of mind.

Methodological and operational limitations inform future directions. The relatively brief cross-sectional nature of this evaluation prevents causal inferences and prospective longitudinal designs are required to isolate the sustained effects of audio reinforcement on engagement and clinical outcomes. While Talkcast is associated with higher platform engagement, we cannot conclude that its use leads to a reduction in symptoms or an improvement in treatment effectiveness. At this stage, the data support the tool's feasibility and acceptability. Future evaluations should include validated clinical outcome measures, such as the Patient Health Questionnaire-9 (PHQ-9) or Generalized Anxiety Disorder-7 (GAD-7) in order to examine therapeutic benefit. Moreover, the feedback data should be interpreted within the context of potential nonresponse bias. Feedback came from only a subset of providers (*n* = 1,103 of 2,791) and clients, so it is possible that individuals who were more technologically engaged or had stronger opinions of the tool were more likely to provide ratings. However, the data did not follow the “U-shaped” distribution of positive and negative responses typically associated with extremity bias (21). Rather, responses were primarily positive. Additionally, technical issues and workflow concerns reveal critical barriers to full adoption that may have artificially suppressed the feasibility metrics. Product improvements, such as the use of AI to edit the Talkcast scripts prior to generation, will also inform future feasibility and acceptability research.

This preliminary evaluation of the feasibility and acceptability of a novel AI-powered tool for between-session client engagement highlights the potential for AI technology to augment therapy sessions and reinforce therapeutic learning. By leveraging AI to automate the creation of personalized, clinician-vetted reinforcement materials in a low-barrier audio format, Talkcast addresses a resource gap that often hinders blended care. These findings highlight the potential for personalized digital tools to bolster treatment adherence and facilitate a more continuous, integrated therapeutic process.

## Data Availability

The datasets generated or analyzed during this study are not publicly available because they were collected as part of standard business operations. Requests to access the datasets should be directed to the corresponding author/s.
